# Supplementary material for: Modelling cell shape in 3D structured environments: A quantitative comparison with experiments
Source: PLoS Comput Biol. 2024 Apr 4;20(4):e1011412. doi: 10.1371/journal.pcbi.1011412 (PMC11020930; doi:10.1371/journal.pcbi.1011412)
Supplement: S1 Appendix — (PDF) [file pcbi.1011412.s001.pdf]

S1 Appendix

To determine the influence of the nucleus on the cell shape, we compare simulated cell shapes without a nucleus, which can be seen in Fig. S1 and Fig. S2 with the simulation results, where we represented the nucleus as a cellular compartment with an elastic volume constraint and interaction energies.

Notably, the simulation without explicit representation of a nucleus results in simulated shapes that are closer to the experimentally observed shapes for the elastic area constraint, compare Fig. 4 and Fig. S1. Visually, a difference can be seen for low elastic area constraint λ_a and interaction energy $J_{\mathrm{medium,cytoplasm}}$, where the removal of the nucleus leads to an invaginated arc instead of a w-shaped spanning. In most other cases, the reduction of Δ_{30} is due to a thickness increase of the cell which was prevented previously due to the high interaction energy penalty between the nucleus and the medium $J_{\mathrm{medium,nucleus}}$.

Similarly, Δ_{30} is below 0.1 for all tested parameters in the simulation without nucleus and with linear area energy, see Fig. S2. The minimum $\Delta_{30} = 0.060$ is found for $J_{\mathrm{medium,cytoplasm}} = 0$, and $\lambda_A = 200$. Here, we find an invaginated arc and full adhesion to the scaffold. In accordance with the simulations with nucleus, we find that the simulated shape best corresponding to the experiment is found for $J_{\mathrm{medium,cytoplasm}} = 0$. Increasing both the interaction energy and the area constraint leads to partial uncovering of the scaffold and evaginated arcs.

The small difference between simulated cell shapes with and without nucleus lead us to the conclusion that for cells in L-shaped structured environments, the cytoskeleton is deforming the spherical nucleus such that it is not a determining factor of the overall cell shape, even though it is much stiffer than the cytoplasm.

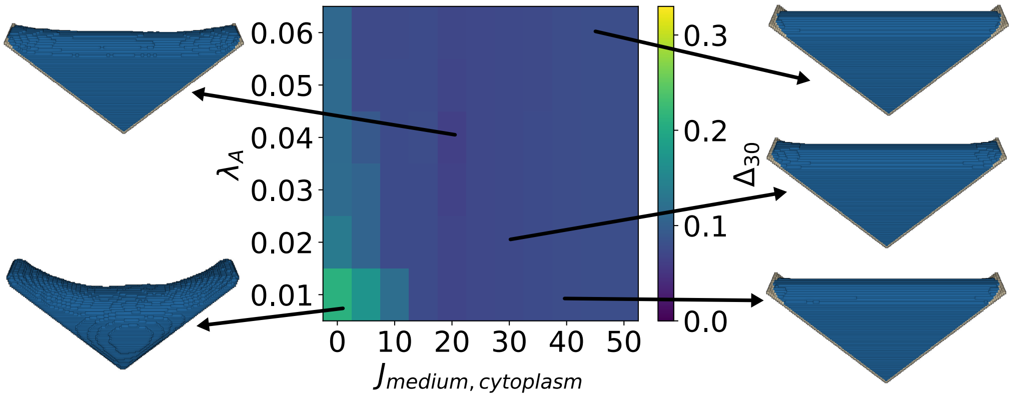

Fig S1. Cell shape difference Δ_{30} between the experimentally observed cell shapes and simulated cell shapes without nucleus obtained with the elastic area energy (Eq. 1) as a function of surface energy constraint λ_A and interaction energy $J_{\text{medium,cytoplasm}}$. Simulated cell shapes for exemplary parameter choices are presented. The minimum cell shape difference $\Delta_{30} = 0.063$ is found for $J_{\text{medium,cytoplasm}} = 20$ and $\lambda_A = 0.04$, the corresponding simulated cell is depicted on the top left.

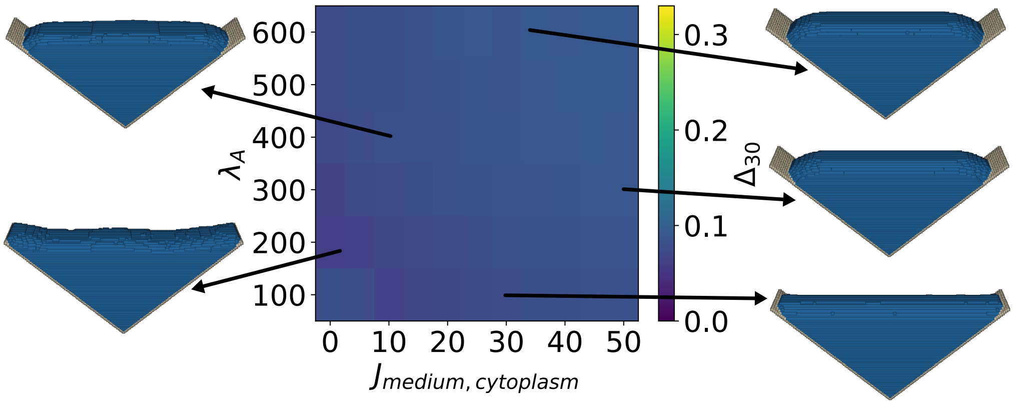

Fig S2. Cell shape difference Δ_{30} between the experimentally observed cell shapes and simulated cell shapes without nucleus obtained with the linear area energy (Eq. 2) as a function of surface energy constraint λ_A and interaction energy $J_{\text{medium,cytoplasm}}$. Simulated cell shapes for exemplary parameter choices are presented. The minimum cell shape difference $\Delta_{30} = 0.060$ is found for $J_{\text{medium,cytoplasm}} = 0$ and $\lambda_A = 200$, the corresponding simulated cell is depicted on the bottom left.
