# Supplementary material for: Modelling cell shape in 3D structured environments: A quantitative comparison with experiments
Source: PLoS Comput Biol. 2024 Apr 4;20(4):e1011412. doi: 10.1371/journal.pcbi.1011412 (PMC11020930; doi:10.1371/journal.pcbi.1011412)
Supplement: S1 Table — (PDF) [file pcbi.1011412.s002.pdf]

Table S1. Parameters for the cellular Potts model simulations.

**Parameter**	**Value**
Monte Carlo Steps	2000
Simulation Temperature	100
Dimensions	140x140x100 vx
$V_{T_{\text{cytoplasm}}}$	36789 vx
$\lambda_{V_{\text{cytoplasm}}}$	0.1
$V_{T_{\text{nucleus}}}$	10890 vx
$\lambda_{V_{\text{nucleus}}}$	1.0
$J_{\text{medium,cytoplasm}}$	0.0 - 50.0
$J_{\text{medium,medium}}$	0.0
$J_{\text{medium,scaffold}}$	0.0
$J_{\text{medium,nucleus}}$	80.0
$J_{\text{cytoplasm,nucleus}}$	10.0
$J_{\text{cytoplasm,scaffold}}$	-50.0
$J_{\text{nucleus,scaffold}}$	5.0
Neighbor Order	7
λ_N	100
