# Supplementary figures and images for: Modelling cell shape in 3D structured environments: A quantitative comparison with experiments
Source: PLoS Comput Biol. 2024 Apr 4;20(4):e1011412. doi: 10.1371/journal.pcbi.1011412 (PMC11020930; doi:10.1371/journal.pcbi.1011412)

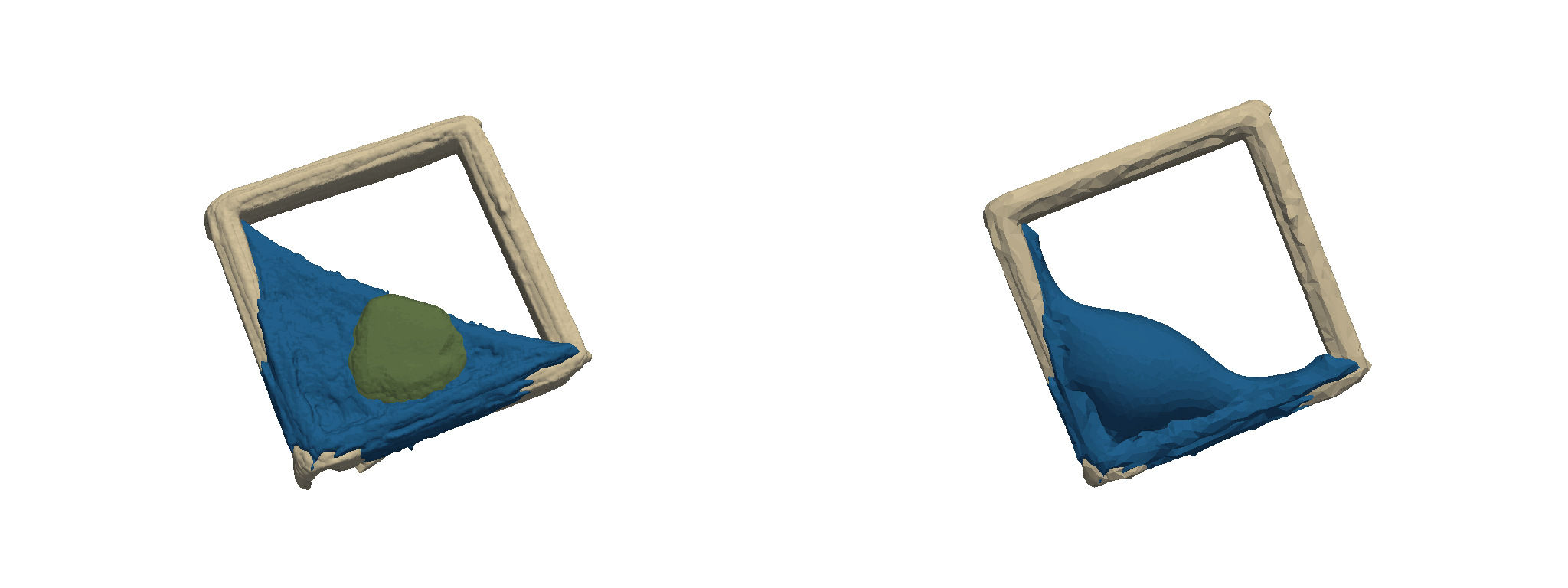

Supplement: S1 Video — (GIF) [file pcbi.1011412.s003.gif]

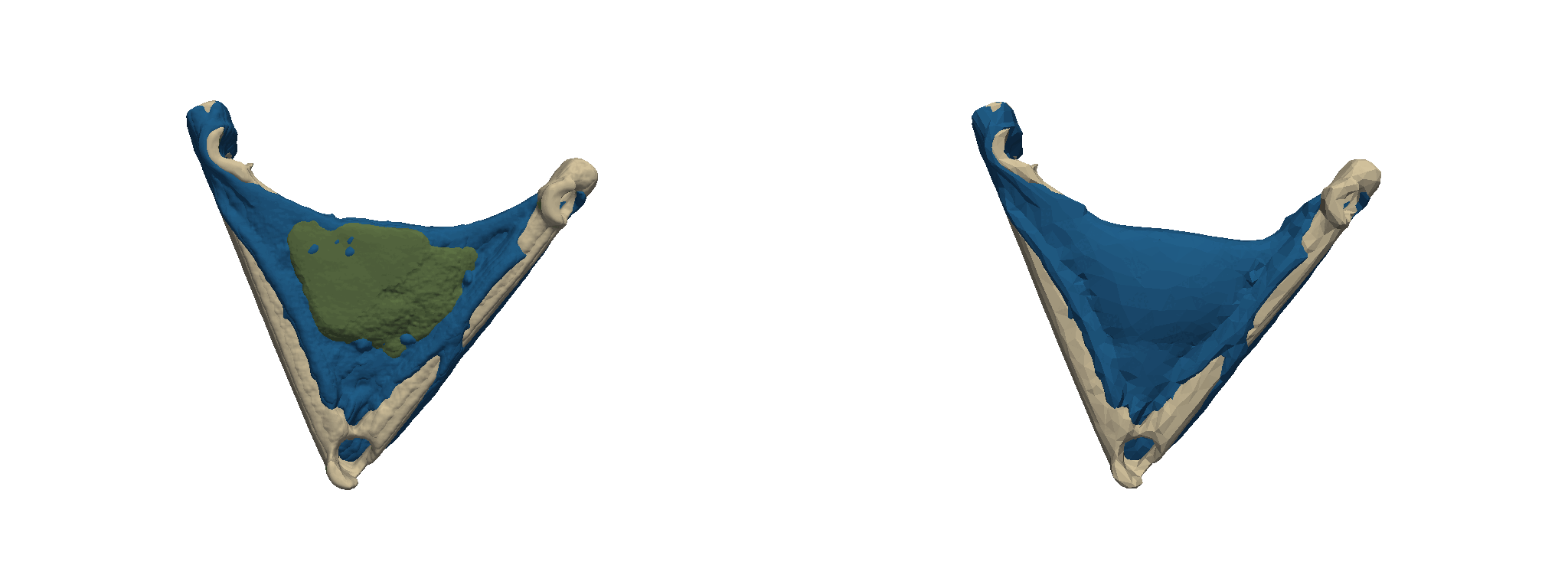

Supplement: S2 Video — (GIF) [file pcbi.1011412.s004.gif]

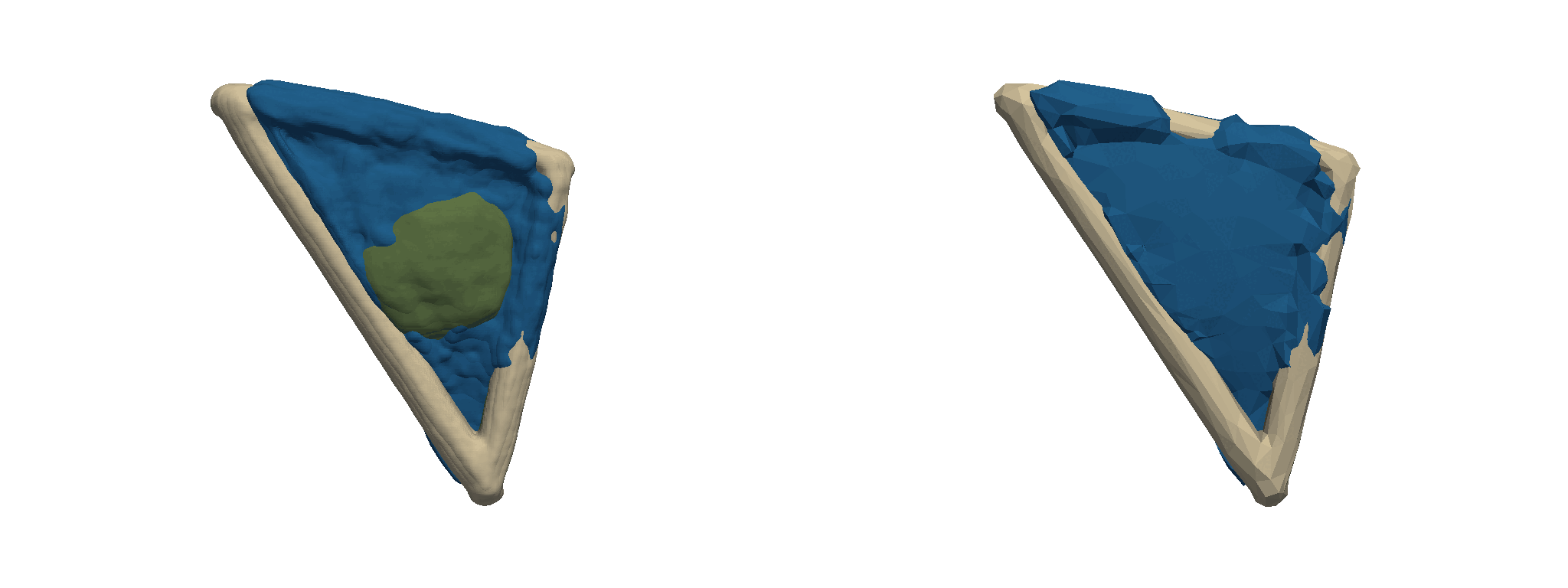

Supplement: S3 Video — (GIF) [file pcbi.1011412.s005.gif]

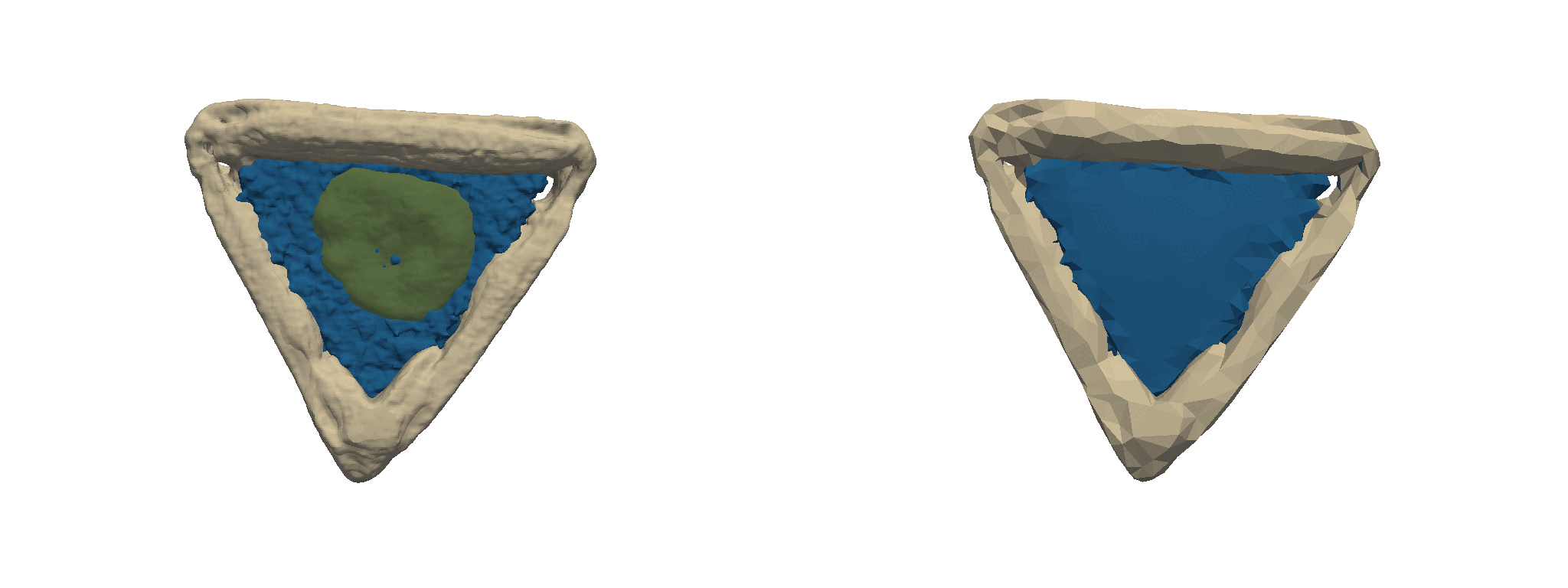

Supplement: S4 Video — (GIF) [file pcbi.1011412.s006.gif]

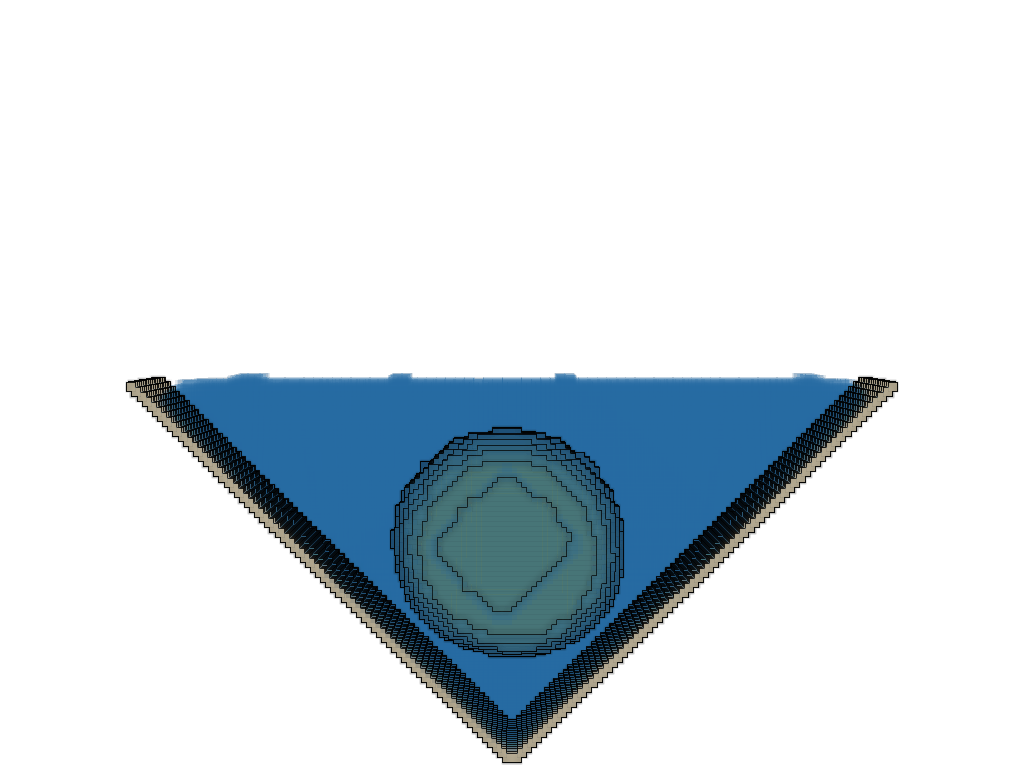

Supplement: S7 Video — (GIF) [file pcbi.1011412.s009.gif]
