# Supplementary material for: Modelling cell shape in 3D structured environments: A quantitative comparison with experiments
Source: PLoS Comput Biol. 2024 Apr 4;20(4):e1011412. doi: 10.1371/journal.pcbi.1011412 (PMC11020930; doi:10.1371/journal.pcbi.1011412)
Supplement: Attachment — Submitted filename: Reply.pdf [file pcbi.1011412.s010.pdf]

Editors

You will find two extensive reviewer reports on your manuscript. Both these reviewers are experts in the topic of the paper and I hope you will find these comments useful in revising your paper. While reviewing your manuscript, I would also request that you pay special attention to the narrative so that the different model assumptions and comparisons are made clear. We look forward to receiving your revision.

We thank the editor for suggesting a revision along the favorable and helpful comments of the two reviewers. We now have put together the code and data for this work and deposited it on zenodo (https://doi.org/10.5281/zenodo.10673295, compare section on code and data availability). We have also included one new figure (Fig. 2) and improved the narrative by rewriting the text throughout. This also resulted in the inclusion of quite a few more references. Finally, we provide a markup copy in which the most essential changes are marked (but not all the detailed ones). We hope that our manuscript can now be accepted for publication in its revised form.

Reviewer #1

In the manuscript entited "Modelling cell shape in 3D structured environments: a quantitative comparison with experiments" by Link et al, the authors use variations of two modeling strategies to reproduce geometries of cells grown in 3D laser printed scaffolds of various shapes. I note to the editor that I cannot comment on the validity or approach of the the experimental methods. The two modeling approaches are to perform surface relaxation to approach a constant mean curvature surface (CMC), and a cellular Potts model (CPM). The authors find that the outputs from surface relaxation using Surface Evolver deviate from the observed geometries. Performing parameter variations for the CPM produces geometries with improved correspondence with the experimental geometries. The modeling efforts appear to be technically sound, although additional care to modeling details and comparisons is warranted. Further discussion on the biological appropriateness of the parameters they identify could improve the strength of conclusions of this work. The work is overall of interest to the readership of PLOS Computational Biology.

We thank the reviewer for this positive assessment and agree that our initial manuscript could be further improved in regard to the mentioned aspects.

Major points:

1. (SI Table 1) What are the units for values in this table?

These are CompuCell3D parameters and we now have specified them with voxels. We note that the CPM-community in its 30-years history has established a standard choice of these parameters that we follow here. The simulation temperature is often set to 100, as done here and now mentioned in the manuscript, and sets the energy scale, which is the central concept of the CPM. Interaction parameters are often chosen in the order of 10, as done here for cytoplasm and nucleus. What is special in our work is the choice of a strongly negative J for the interaction between cell and scaffold. This choice is usually not required when studying e.g. differential adhesion, but here we use it, similar to our earlier work for cell adhesion on 2D patterns (Vianay et al. PRL 2010, Albert and Schwarz BPJ 2014).

2. How do the best fit parameters for the CPM model compare against experimental properties? Is there anything to be learned from the strength of adhesions, surface tension, etc.?

Our main point is that surface tension alone is not sufficient to predict cell shape. Surface tension can explain many shape features of cells, including the invaginated arcs often seen when cells adhere to a few localized adhesions. We have analyzed these arcs before also for cells in 3D scaffolds with point-like adhesions (Brand et al. BPJ 2017), but at that time, we did not consider the full cell shape. In addition, now our scaffolds are completely coated with FN, to achieve strong interactions between cells and scaffolds. We now segmented and analyzed the full cell shape for these scaffolds and found that surface tension alone is not enough to explain it. Note that for the tension model, an explicit energy scale is not required, because there is only one.

We agree with the referee that it would be instructive to convert our CPM-results to more physical statements, but in our understanding, this is not possible in a straight-forward manner. In the seminal work by Magno et al. BMC Biophysics 2015, it has been shown that the CPM agrees well with the main conclusions from other cell surface mechanics models, including explicit surfaces and the vertex model. However, it also has been shown that there is no simple mapping between these models, that the results of the CPM depend on the level of resolution (pixel size) and that the CPM works best with a neighborhood summation, which is also a main aspect of the results presented here. In our understanding, it is the combination of a more complicated surface Hamiltonian and the neighborhood summation that leads to the better agreement with the experimental data demonstrated here. At the same time, this makes it difficult to give precise numbers. The most naïve estimate would be to say that J/a^2 should be surface tension (interaction parameter J, pixel size a), which is known to be around 1 nN/μm (compare e.g. Bischofs et al., PRL 2009). Assuming a cell size of 10 μm and correspondingly a pixel size of around 1 μm in our simulations, the energy scale T=100 would correspond to 100 nN μm, which makes sense because we know that 5 nN and 1 μm are exactly the forces and dimensions at focal adhesions, and there are several focal

adhesions involved here. With these values, all model parameters would have physical dimensions. However, it is hard to estimate the effect that neighborhood size N has on these values. A more detailed analysis would require simulations with different resolutions, which is not the focus of this work, so we keep with the tradition in the CPM-community and prefer not to convert into numbers.

In the introduction and the discussion, we now introduce our understanding of the CPM in more detail and make clear that all parameter choices are very reasonable and in line with earlier work. We also comment more on the choice of parameters in Tab. 1. We have included the simple estimate of the energy scale into the discussion and write that a more detailed analysis needs simulations with different resolutions.

3. (Line 176) Since the simulation is stochastic monte carlo, the system will tend towards lower energy states but there will be fluctuations around a local minima (c.f. supplemental movies). Moreover, depending on the energy landscape and the magnitude of fluctuations, a system can be kinetically trapped and thus prevented from reaching the "lowest energy state". The statement should be amended accordingly.

While we acknowledge that kinetic trapping is possible in principle, we believe that for the relatively simple shapes considered in our simulations, kinetic trapping is unlikely. Simple shapes exhibit smoother energy landscapes with fewer local minima, making it easier for the system to explore and find the global minimum. We now explain this aspect on line 196. In the discussion, we now also mention that in the future, more physical dynamics could be used, including a very recent suggestion for a CPM-algorithm that is motivated by stochastic thermodynamics, obeys detailed balance and represents non-equilibrium physics (Belousov et al. arXiv 2023).

4. Related to major point 3, how was the representative output of each CPM simulation selected? The minimal energy configuration observed from the trajectory?

Because our CPM-simulations work at constant temperature and lead to surfaces with roughness, we follow the standard procedures for Monte Carlo simulations and average over a large number (500) of configurations after reaching steady state, as now explained on line 238. Alternatively, one could lower temperature and the shape would become smoother, as done before for cell shape on 2D micropatterns (Vianay et al. PRL 2010), as now stated in line 198.

5. (Section Comparison to CPM-Shapes) The initial configuration of the cellular Potts model is not clear. From the supplemental movies, it appears that an idealized scaffolding structure is used. For quantitative comparison using Δ30 against the CMC results, it would be beneficial to use similar inputs and constraints. Alternatively differences between workflows should be stated and possible limtations discussed.

As explained above and now also in the manuscript, for the relatively simple shapes considered here, initial conditions do not matter much. We used triangular prism pyramid occupying the space between the adhesive struts, as explained on line 236, but other shape would lead to the same results. The initial shapes used here can also be seen in movies 5 and 6.

6. What are the reported differences between CPM and experimental geometries. Are they also mean Δ30 values as reported for CMC results?

We use Δ_{30} throughout our work. However, as the reviewer noted, there is a difference between the CMC- and CPM-cases. For the CMC-cases, every surface minimized for surface energy is compared to its initial experimental shape and this results in a Δ_{30} value. For the CPM-cases, we compare the mean shape obtained from the simulations to the mean frequency spectrum obtained from all experimental examples in the L-class. For cells in L-shaped scaffolds, the difference Δ_{30} between the spectrum of the CPM-shape and the averaged spectrum of the experimental examples is for most parameters smaller than the difference between the minimized surface and the experimental shape, which leads to the conclusion that the CPM performs better. We are sorry that his information was missing in the initial submission and now explain this procedure better in the CPM-case (line 397 onwards), especially the procedure of using an averaged spectrum to represent the experimental data.

7. Uncertainty quantification of Δ30 should be shown where possible for modeled geometries against experiment. If possible, statistical hypothesis tests could also be used to guide interpretation of Δ30 differences.

The hot plots from Figs. 5-7 provide a measure for the variations of Δ_{30} with CPM-model parameters. But it is true that we did not provide a good reference of Δ_{30} in general. We now have conducted a computation to show which values result when approximating a sphere by an ellipsoid. We have added a new Figure 2a, which shows Δ30 values between a sphere and the ellipsoid with same volume, but different aspect ratio. Typical values for experiments versus minimized shapes are around 0.1, which according to these results corresponds to an aspect ratio below 1.5. These results now are used as reference for the rest of the manuscript. We thank the reviewer for this very helpful question.

8. Establishing a baseline or threshold for Δ30 values may also be helpful for interpreting the modeling results. For example, an NxN heatmap of Δ30 for experimental geometries of cells grown in a common scaffold against each other could give a sense of variability in shape from cell to cell. Further it would be beneficial to show results from several cells or models.

We thank the reviewer for this excellent suggestion and now have computed Δ_{30} values between experimental data sets. We have added a new Fig. 2c, which shows that the experimental shapes differ significantly for the L-scaffolds (where we have the largest number of data sets). In particular, they are larger than the Δ_{30} we find for CPM-shapes for a large parameter range. One reason for this large variance is the height differences for the cells in the L-shapes, as demonstrated in Fig. 2b. We therefore have ordered the examples from Fig. 2c according to their height, which generates more structure in this plot.

9. (Data availability) Imaris, a commercial software, is used to extract triangulated meshes. It would benefit reproducibility to share the output triangulations, input micrographs, and segmentations.

We now have deposited the triangulation data obtained by Imaris on zenodo as WRL-files. The input files (Zeiss image files) are too large to be deposited, but can be obtained from the authors upon request. Additionally, we deposited the python notebook that can be used to calculate Δ_{30} from the WRL-files. WLR-file can be converted to STL-files with MeshLab (https://www.meshlab.net) and we also provide a script to convert STL-files to FE-files for use with SurfaceEvolver.

Minor points:

1. (Line 325) Table S1 is missing “S1"

We have corrected this.

2. (Discussion) The authors suggest that their approach could be used for rational scaffold design despite that they found limited transferrability of parameters from scaffold to scaffold. I think some discussion of biological correspondence of their parameters and/or what may be missing from the model to lead to universal parameters could benefit their claim.

As also commented by reviewer #2, the statements on rational design were confusing and we have removed them. In the last paragraph of the discussion, we now give a more in-depth explanation of how we interpret our results from the biophysical point of view. Our main conclusions are: (1) surface tension is not enough to predict cell shape if cells adhere strongly to scaffolds; (2) the CPM represents more physical effects, including bulk terms, and therefore performs better; (3) the fact that the linear area constraint works better than the elastic one suggests that surface area is not strongly regulated, but created as adhesion increases; (4) the fact that modelling the nucleus does not matter much suggest that it is strongly anchored in the middle of the cell by the cytoskeleton and thus kept away from the cell surface. We believe that these conclusions are very interesting in themselves. We note the strength of the CPM in obtaining these results, even if we cannot convert its parameters into specific physical numbers at this point.

Reviewer #2

Summary: This paper describes a systematic comparison of cell shapes grown in 3D structured environments with different modeling approaches – an energy-minimizing Constant Mean Curvature (CMC) surface model, and different variants of a cellular potts model (CPM). They analyze the 3D shapes with a 3D spherical harmonics analysis to get the frequency spectra that can be directly compared between experiment and model. Comparison with experiment demonstrates that the CPM area energy approach captures the shape better. There is some discussion of the 3D scaffold production, and the rational design of scaffolding in the future, but this reviewer thinks it muddles the more obvious narrative of the paper. While the narrative can be improved, the content of the paper is exciting and worthy of publication after minor revisions.

We thank the reviewer for this positive assessment and agree that the manuscript could be improved in regard to the narrative.

Specific comments:

Overall:
- The description of the 3D scaffold production with laser nano-printing seems over-emphasized in the abstract/introduction and should be framed more as supporting information for the shape analysis. The main concern of the 3D scaffolds should be why these particular four shapes were chosen and how they influence the resulting cell shape analysis.

We agree that this aspect was overemphasized and now have changed the narrative to focus more on the biophysical messages. We have removed references to laser writing from abstract and author summary, where we now only mention that we investigate cell shape in 3D scaffolds. In the main text, we have extended introduction and discussion towards more biophysics, and improved the description of our results in the main text. This being said, however, we kept the description of the experimental procedures, because this is an integral part of our work and needs to be documented, so that it can be repeated by others.

- The analysis, which exploits the particular scaffold shape for simplifying the coordinate system, should also be better justified. Are these regular-shaped scaffolds over-simplifying the shape of cells which would likely be much more complex in natural environments?

We certainly believe that cell shapes in 3D tissue are more complex. For an example of shape analysis of highly branched endothelial cells in connective tissue, we refer e.g. to the work by Claire Waterman and Gaudenz Danuser (Elliot et al. NCB 2015). The shapes used in our work on 3D laser-printed scaffolds were selected because they are especially suited for quantitative analysis with a standard confocal microscope. They were motivated by the 2D shapes pioneered by Manuel Thery, who in his PhD-work considered triangles, V, arc and tripod shapes in one sequence because the number of invaginated arcs increases from zero to three (Thery et al. Cell Motility and the Cytoskeleton 2006). Later his group introduced other shapes, like the L-shapes which are very prominent in our work. The main idea of the current work was to keep the cells close to the substrate, because this facilitates cell seeding and imaging, but to avoid complicating features like the middle pillar used in our earlier work on arc analysis in 3D scaffolds (Brand et al. BPJ 2017). This explains the choice of structures seen in Fig. 1 and is now explained better on lines 90-97.

For a general discussion of cell shapes in different environments, we refer to our recent review on this subject (Link et al. Adv. Funct. Mat. 2023). There we argue that even in complex environments, cells show the same shape elements, such as invaginated arcs, although in general, mesenchymal cells in biological tissue typically show more branched morphologies due to the porous nature of connective tissue. Therefore, the simple structures used in this work should be sufficient for our purpose, as we focus on the underlying physical principles.

In the introduction, we now explain better the biological complexity of different cell types and that here we focus on mesenchymal cells in structured environment, which however lead to the same shape elements as seen in real tissue.

- Since the main purpose of this paper is to compare experiments with models, it should focus more explicitly from the start on comparing the different model methods. It would make for a stronger narrative if there was a more systematic introduction to the modeling approaches, their assumptions, and their algorithms.

We agree and have worked on the narrative. In particular, we now explain the CPM in more details at different parts in the manuscript.

Abstract:
- “3D scaffolds for cell adhesion were manufactured using 3d laser nano-printing and the shapes of single mesenchymal NIH/3T3 cells were compared by a Fourier method with surfaces that minimize area …” This makes it sound like the 3D scaffolds are compared with the shapes of the cells. Please reword more carefully, separating out the different important concepts.

Modified as suggested.

- “Rational design of scaffolds for desired cell shape” Is this paper more about designing the scaffolds, or making conclusions about cell shapes by comparison with modeling? The way this is introduced in the abstract will influence the reader’s evaluation of this paper.

We agree that design is not the subject of this work and have removed this sentence.

3D structured environments:
- Add labels for the four shapes in Fig 1a to make these names less ambiguous when reading the text. “L-shaped” and “V-shaped” with “right triangle” or “equilateral triangle” are currently used, please reuse these same names throughout the text.

Modified as suggested.

Constant Mean Curvature (CMC) surfaces:
- This needs to be more clearly introduced as one of the modeling approaches when described in methods. As it is currently written, it can easily be interpreted as a step of the analysis.

Modified as suggested.

Cellular Potts Model (CPM):
- I would like to see a more explicit treatment of the different variants of CPM used. Currently these are addressed in one long paragraph. This needs at least one paragraph for the elastic energy assumptions and model, and one for the linear area energy assumption and model. It would be useful to assign these explicit names, so they can be referenced later in the paper. Also mention how many simulations were run, what were the parameters, and any other important metadata if anyone wants to reproduce these simulations.

We have rephrased this paragraph to emphasis the differences between the models.

- “Cellular Potts models (CPMs) are a more evolved approach to model single cells” What do you mean by “more evolved”?

CPMs are more evolved as in its 30 years of existence, the community has branched out in many different directions, which is also reflected by the different software tools which are now available (including CC3D used

here, but other large packages such as Mephisto or Aristoo). To avoid confusion, though, we now have removed this phrase.

3D Spherical Harmonics Analysis:
- These particular scaffold shapes, combined with the spherical harmonics analysis, allow the authors to exploit the star-shapes to map to a simplified unit sphere. This seems like it might be oversimplifying the analysis of cell shape to a very restricted domain.

We agree that this approach would not work in the general case, for example for the branched morphologies analyzed in Elliot et al. NCB 2015, but here we have specifically only chosen shapes which lead to star-shaped shapes. Fourier methods have been used a lot before in 2D and we now cite corresponding reviews by the groups of Julie Theriot and Rob Murphy in the introduction. They are a natural choice because they are motion-invariant. Moreover, they fit well to our CPM-approach, which cannot describe features smaller than the lattice constant, thus there is a natural cutoff for a Fourier spectrum. The same holds true for the imaging data due to the finite optical resolution. We now include such a statement in the main text.

- Please describe why you chose to use the first 30 degrees of the respective frequency spectra. What kinds of shape features do you expect to detect in this range?

30 was chosen by visual inspection and seems more than enough for the data at hand. Note that both the imaging data and the CPM-simulations have natural cutoffs (optical resolution and lattice size). Actually 15 would be sufficient as seen in Fig. 3 and we chose to double this number.

Results:
- Fig 2. Call this something more obvious like "Comparison between experimental cell shapes and minimized surface model"

Modified as suggested.

Data availability

Reviewer #1: **No:** The authors note that scripts and data will be made available on Zenodo upon publication. It is not clear if the input experimentally derived cell shapes nor raw output data are included in the deposition.

We now have deposited the data in public mode on zenodo before publication, so that the reviewers can look at them. We do not provide the Zeiss data files, but all WRL-files obtained by image processing with Imaris. We also included all scripts used for data analysis and simulations as explained on the zenodo page (https://doi.org/10.5281/zenodo.10673295).
